# Marriage and Cancer Risk: A Contemporary Population-Based Study Across Demographic Groups and Cancer Types

**DOI:** 10.1158/2767-9764.CRC-25-0814

**Published:** 2026-04-08

**Authors:** Paulo S. Pinheiro, Amber N. Balda, Hannah M. Cranford, Tracy E. Crane, Erin N. Kobetz, Frank J. Penedo

**Affiliations:** 1 Sylvester Comprehensive Cancer Center, University of Miami School of Medicine, Miami, Florida.; 2 Division of Epidemiology and Population Health Sciences, Department of Public Health Sciences, University of Miami School of Medicine, Miami, Florida.

## Abstract

Marriage is consistently associated with earlier cancer diagnosis and improved survival, but its relationship to cancer incidence is less understood. We examined cancer risk by marital status across cancer sites, sex, race/ethnicity, and age using large-scale, population-based US data. We analyzed Surveillance, Epidemiology, and End Results data from 12 states for adults ages ≥30 years, with denominators from the American Community Survey (2015–2022). Age-adjusted incidence rates were calculated, and negative binomial regression estimated incidence rate ratios (IRR) comparing never-married with ever-married adults, stratified by sex, age group, and cancer site. Never-married adults had higher cancer incidence [IRR = 1.68; 95% confidence interval (CI), 1.53–1.84 in men; 1.85; 95% CI, 1.68–2.03 in women] across nearly all major cancer sites and racial/ethnic groups (IRR range: 1.62 in White men to 1.96 in Black men). Never-married Black men had the highest incidence; among ever-married men, Black men had lower rates than White men. Among men, site-specific IRRs reached 5.04 for anal cancer; among women, 2.64 for cervical cancer. Pronounced marital disparities were observed for gynecologic cancers and for cancers linked to infections, tobacco, and alcohol, whereas differences were smaller for breast, thyroid, and prostate cancers. Ever-married adults consistently showed lower cancer risk. Whereas some variation may reflect selection into marriage, the magnitude and site-specific patterns of association suggest that marital status stratifies cancer risk through cumulative social and behavioral pathways, including those relevant to infection-related and lifestyle-associated cancers.

**Significance:**

Ever-married status may serve as a useful social indicator for cancer risk stratification and prevention.

## Introduction

Marriage has long been associated with better health outcomes, including lower morbidity, longer life expectancy, and more favorable self-rated health (1–6). These benefits are thought to arise from increased social support, healthier behaviors, and greater economic stability among married individuals. Moreover, never-married individuals tend to exhibit more adverse physiologic profiles by mid-life, including elevated inflammation and metabolic dysfunction (7).

However, the health benefits of marriage are not evenly distributed. Men tend to benefit disproportionately from health and social advantages associated with marriage, whereas effects for women are more variable (7, 8). When considering race, the benefits of marriage seem strongest among White men, whereas they are more modest or inconsistent for White women and Black adults (6). This reflects structural inequities in how social and economic advantages of marriage are accessed and distributed (4, 9).

In oncology, prior research on marital status has focused almost exclusively on postdiagnosis outcomes. Previous studies have shown that married patients with cancer are more likely to be diagnosed at earlier stages (10, 11) and experience higher survival compared with unmarried individuals (12, 13). These advantages are often attributed to spousal support in promoting screening, enhancing treatment adherence, and navigating complex care (14–16). Yet, little is known about whether marriage influences the risk of developing cancer, an important and understudied question.

To date, only a few population-based studies have directly examined cancer incidence by marital status. One study from Michigan (1978–1982) found higher incidence among never-married Black adults (17). Another from Japan (1980–1984) reported elevated risk for single adults across multiple cancer sites (18). A third analysis, using data (1969–1971) from the US Third National Cancer Survey, also found differences by marital status, race, and sex (19). However, these studies are outdated or based on small, regional populations from more than 40 years ago. Since then, most research has relied on hospital-based samples, case–control designs, or secondary analyses of clinical trial data. These approaches lack population denominators and are vulnerable to selection bias. Furthermore, earlier studies predate major shifts in marriage patterns, screening practices, and exposures such as obesity, smoking, and human papillomavirus (HPV), limiting their contemporary relevance (20–23).

This question is increasingly important considering declining marriage rates, delayed childbearing, and shifting norms around partnership and family formation (22–25). Marriage reflects enduring social, economic, and institutional ties that may influence cancer risk through multiple mechanisms. Potential protective effects include healthier behaviors such as reduced tobacco and alcohol use, more favorable sexual and reproductive patterns, greater stress resilience, and improved access to preventive care, including cancer screening (14, 26–34). Notwithstanding these protective factors, individuals who marry may already differ from those who do not in ways that affect cancer risk. Selection factors such as better baseline health, lower substance use, higher income, and stronger social integration may predispose individuals both to marry and to have lower cancer risk, complicating the interpretation of the association between marital status and cancer risk (35–38).

In this study using nationally representative data, we offer the most comprehensive and contemporary analysis to date of how cancer incidence varies by marital status across demographic groups and cancer types.

## Materials and Methods

### Data source and study population

We analyzed 8 years of data (2015–2022) from 12 Surveillance, Epidemiology, and End Results (SEER) participating states selected based on the availability of individual-level data on marital status at the time of diagnosis: California, Connecticut, Georgia, Hawaii, Idaho, Iowa, Kentucky, Louisiana, New Jersey, New Mexico, New York, and Utah. Data were obtained from the SEER Research Plus 17-Registries database (RRID: SCR_006902). This dataset includes other variables such as age, sex, race/ethnicity, tumor stage, and cancer site. In 2022, the combined population of these states represented 103.7 million individuals (39), 31.1% of the US population, encompassing all major racial/ethnic groups.

We included all malignant cancers diagnosed at age ≥30 years, corresponding to the approximate mean age at first marriage in the United States. The study period begins after the 2015 US Supreme Court decision in *Obergefell v. Hodges*, which legalized same-sex marriage nationwide and established a consistent legal definition of marital status across all states.

We utilized SEER’s race/ethnicity recode variable, including non-Hispanic White, non-Hispanic Black, Hispanic (any race), Asian/Pacific Islander, American Indian/Alaska Native (AI/AN), and other/unknown. For ease of presentation, we refer to these groups as White, Black, Hispanic, and Asian/Pacific Islander throughout the article. Due to smaller numbers and known racial misclassification in cancer registry data when Indian Health Service linkages are not available, AI/AN individuals were not analyzed as a separate group (40).

### Exposure definition

We classified marital status at diagnosis as never-married versus ever-married, with the latter group including married, separated, divorced, or widowed individuals. We defined primary exposure as never-married versus ever-married at diagnosis, a prespecified, policy-relevant contrast reflecting legal marital status as a social and institutional classification. This binary definition minimizes misclassification relative to attempting to reconstruct marital trajectories (e.g., divorce, widowhood, and remarriage) that are simply not collected in population-based datasets such as SEER and the American Community Survey (ACS). Given the interest of the exposure as marriage *per se*, individuals who were partnered or cohabiting but not legally married were classified as never-married, consistent with that exposure definition and the structure of SEER and ACS data. Ever-married individuals served as the reference group in all regression models.

### Cancer classification

Cancer sites were classified using SEER site recode International Classification of Diseases for Oncology, Third Edition (ICD-O-3)/World Health Organization 2008 definitions, consistent with the Annual Report to the Nation (41). Additional classifications were applied for selected cancers. Breast cancer subtypes were defined by hormone receptor (HR; estrogen or progesterone receptors) and HER2 receptor status (42). Hepatocellular carcinoma (HCC) was defined using ICD-O-3 site/histology codes C22.0 and 8,170 to 8,175 (43) to distinguish it from intrahepatic cholangiocarcinoma. Prostate cancers were grouped by prostate specific antigen (PSA) level at diagnosis into three categories: PSA1: <10 ng/mL, PSA2: 10 to <20 ng/mL, and PSA3: ≥20 ng/mL, reflecting clinical risk stratification.

### Population denominators and rate standardization

Population denominators for incidence rates for each state and calendar year (2015–2022) were obtained from the US Census Bureau’s ACS (RRID: SCR_024727) and pooled across states and years. Denominators were stratified by marital status, 5-year age groups beginning at age 30 years (final category for ages 85+), sex, and race/ethnicity to align with SEER case classifications. All incidence rates were age-standardized to the 2000 US standard population using the direct method.

### Missing data and multiple imputationss

Marital status was missing for 8.1% of cancer cases. To address this and avoid underestimating incidence rates by marital status, we used multiple imputations, generating 10 imputed datasets using logistic regression models that included age, sex, race/ethnicity, state, and calendar year as predictors.

### Statistical analyses

Incidence rate ratios (IRR) comparing never-married with ever-married individuals were estimated using negative binomial regression models to account for data overdispersion. All models included log person-years as an offset and were adjusted for 5-year age group, sex, and race/ethnicity. As a secondary analysis, we stratified by age group (30–54 vs. ≥55 years) to explore the potential distinction between selection effects, which may be more prominent at younger ages when individuals are entering or avoiding marriage, and cumulative health and social benefits of being married, which may emerge at older ages. We also conducted stratified analyses by sex and race/ethnicity and calculated age-adjusted incidence rates by SEER summary stage (localized, regional, and distant) for six common screenable cancers.

To evaluate whether associations varied by sex, race/ethnicity, or cancer site, we introduced two-way interaction terms between marital status and each variable in separate models and compared model fit using likelihood ratio tests. Degrees of freedom reflected additional parameters; tests were two-sided with significance at *P* < 0.05. Model fit and goodness of fit were assessed using deviance, Pearson *χ*^2^ statistics, and information criteria, including the Akaike Information Criterion and Bayesian Information Criterion. Analyses were conducted using SPSS v31.0 (RRID: SCR_002865) and SAS v9.4 (RRID: SCR_008567).

## Results

Table 1 summarizes the annual average population denominators, total cancer case counts, and median age distributions by sex, race/ethnicity, and marital status for adults ages ≥30 years in the 12 SEER states. Overall, between 2015 and 2022, data from 12 SEER selected states captured more than 500 million person-years at risk among individuals ages 30 years and older, representing an annual population of more than 62 million. Overall, 19.2% of this population were never-married (21.5% of men and 17% of women), with substantial variation by race/ethnicity and sex (Table 1). In females, never-married status was most prevalent among Black women (33.8%) and least common among White (12.2%) and Asian/Pacific Islander (13.2%) women; 21.8% of Hispanic women were never-married. The proportion never-married ranged from 17.4% in White men to 34.6% in Black men, with intermediate values in Hispanic (26.8%) and Asian/Pacific Islander men (18.1%).

**Table 1.** Annual average population size, cancer cases, and median age by sex, race/ethnicity, and marital status among adults ≥30 years old.

*N* (%)	Male		Female	
	Never-married	Ever-married	Never-married	Ever-married
**Population annual averages** ^**a**^	​	​	​	​
All combined^b^	6,469,659 (100)	23,676,697 (100)	5,525,691 (100)	26,954,847 (100)
White	2,910,356 (45)	13,858,409 (58.5)	2,172,059 (39.3)	15,566,569 (57.8)
Black	1,158,973 (17.9)	2,191,988 (9.3)	1,347,841 (24.4)	2,645,705 (9.8)
Asian/Pacific Islander	614,445 (9.5)	2,777,884 (11.7)	519,714 (9.4)	3,425,294 (12.7)
Hispanic	1,705,881 (26.4)	4,662,059 (19.7)	1,417,699 (25.7)	5,099,472 (18.9)
Median age	40	55	42	56
Married	—	18,778,990 (79.3)	—	17,878,276 (66.3)
Separated	—	624,817 (2.6)	—	880,982 (3.3)
Divorced	—	3,306,175 (14)	—	4,689,008 (17.4)
Widowed	—	966,715 (4.1)	—	3,506,581 (13)
**Cancer cases (total)** ^**c**^	​	​	​	​
All combined^b^	383,679 (100)	1,763,209 (100)	395,036 (100)	1,698,489 (100)
White	223,209 (58.2)	1,267,625 (71.9)	203,971 (51.6)	1,179,805 (69.5)
Black	78,785 (20.5)	159,438 (9)	85,764 (21.7)	143,463 (8.4)
Asian/Pacific Islander	17,048 (4.4)	118,342 (6.7)	25,948 (6.6)	143,925 (8.5)
Hispanic	55,409 (14.4)	184,588 (10.5)	72,169 (18.3)	209,185 (12.3)
Median age group	60–64	65–69	60–64	65–69
Married	—	1,412,330 (80.1)	—	1,037,777 (61.1)
Separated	—	22,922 (1.3)	—	25,477 (1.5)
Divorced	—	179,847 (10.2)	—	237,788 (14)
Widowed	—	148,110 (8.4)	—	397,446 (23.4)

a Population denominators derived from the ACS, 2015–2022.

b All combined race category includes individuals of other or not specified racial/ethnic groups.

c Cancer incidence data obtained from SEER 12 states combined, 2015–2022.

**Figure 1.**
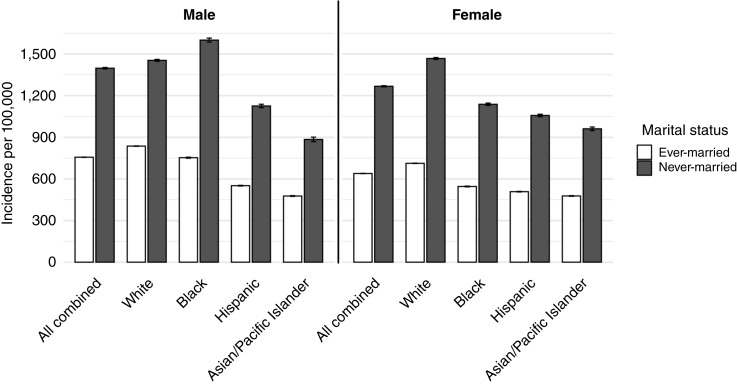
Age-adjusted incidence rates of all cancer combined in individuals ≥30 years old by race/ethnicity, marital status, and sex, SEER 12 states combined, 2015–2022. Note: Error bars represent 95% CIs. Due to the large study population and resulting precision of the estimates, the CIs are extremely narrow and closely overlap the point estimates.

**Figure 2.**
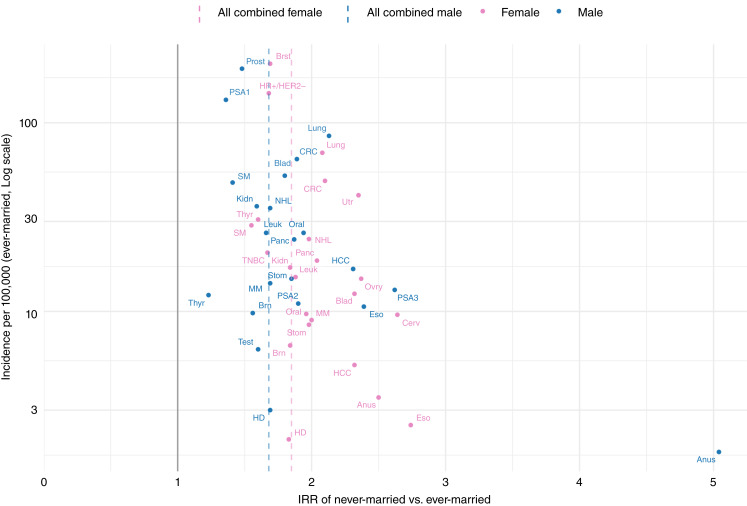
Age-adjusted incidence and IRR of never-married vs. ever-married by sex and cancer site, SEER 12 states combined, 2015–2022. Cancer incidence was significantly higher among never-married adults (IRR = 1.68; 95% CI, 1.53–1.84 in men; IRR = 1.85; 95% CI, 1.68–2.03 in women). Cancer site abbreviations: Brn, brain; Brst, breast; Cerv, cervix; CRC, colorectal cancer; Eso, esophagus; HD, Hodgkin lymphoma; Kidn, kidney; Leuk, leukemia; MM, multiple myeloma; NHL, non–Hodgkin lymphoma; Oral, oral cavity; Ovry, ovary; Panc, pancreas; Prost, prostate (overall); SM, skin melanoma; Stom, stomach; Test, testicle; Thyr, thyroid; TNBC, triple-negative breast cancer; Utr, uterine.

**Figure 3.**
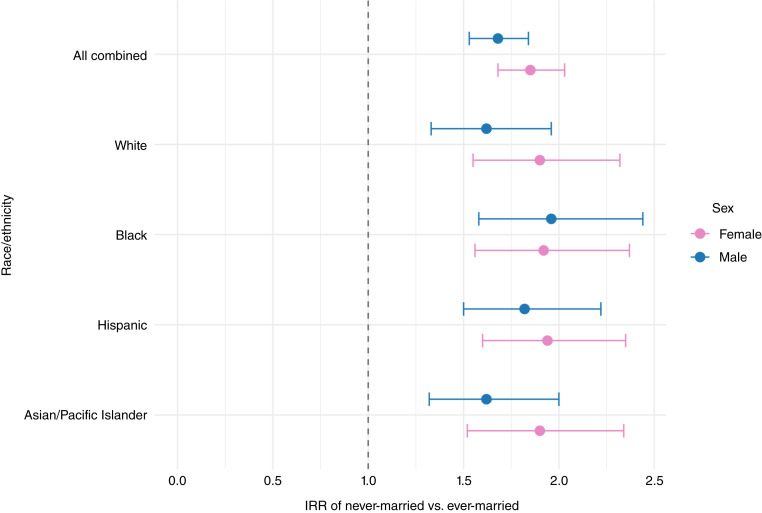
IRRs for never-married vs. ever-married by race/ethnicity and sex, SEER 12 states combined, 2015–2022.

**Figure 4.**
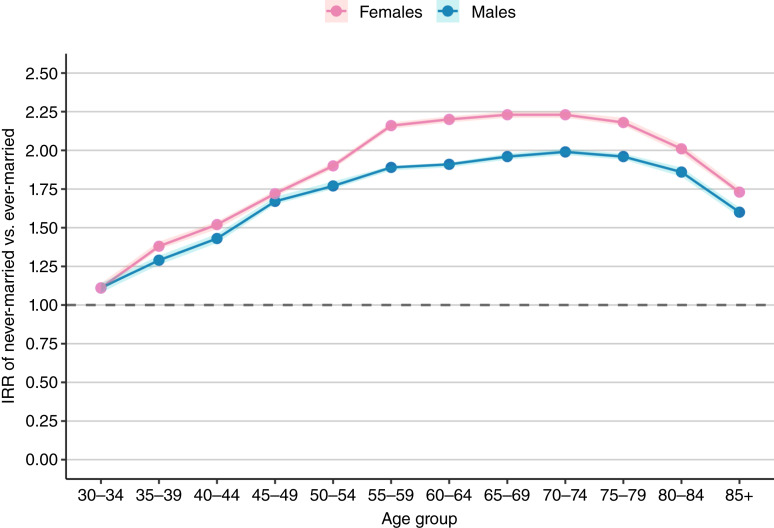
Age-specific cancer risk ratios of never-married vs. ever-married by sex, SEER 12 states combined, 2015–2022. The age-specific IRR for those ages 55 years and older was 1.99 (95% CI, 1.80–2.20), which was higher than the IRR for adults ages 30–54 years (IRR = 1.49; 95% CI, 1.20–1.70). Note: The shaded bands represent the 95% CIs. Given the large number of cases, person-years, and resulting precision of the estimates, the intervals are extremely narrow and closely overlap the point estimates.

During the study period, of 4,240,413 cancers diagnosed among adults ≥30, 18.4% occurred in never-married individuals (Table 1). Across all cancer sites and in both sexes, never-married adults had significantly higher age-adjusted incidence rates than ever-married individuals (Fig. 1; Supplementary Table S1). Among men, the IRRs comparing never- with ever-married was 1.68 [95% confidence interval (CI), 1.53–1.84]; among women, it was 1.85 (95% CI, 1.68–2.03; Fig. 2; Supplementary Table S2). These elevated risks were consistently observed for never-married individuals across all major racial/ethnic groups (Fig. 3; Supplementary Table S3), age categories (Fig. 4; Supplementary Table S4), and all stages at diagnosis (Supplementary Fig. S1; Supplementary Table S5).

IRRs increased with age in both sexes, peaking at 70 to 74 years [1.99 (95% CI, 1.97–2.01) for men and 2.23 (95% CI, 2.21–2.25) for women]. In the 75+ age group, the advantage for ever-married adults lessened for both sexes (Fig. 4). IRRs remained significantly higher in women than in men across all age groups, except for ages 30 to 34 years. When stratified into broader age categories ages 30 to 54 and ≥55 years, the IRRs were 1.49 (95% CI, 1.20–1.70) and 1.99 (95% CI, 1.80–2.20), respectively.

By race/ethnicity, never-married Black men show the highest cancer rates of all groups at 1,600.6 per 100,000 (Fig. 1). However, among married men, Black men at 752.6 per 100,000 had significantly lower rates than married White men with 836.2 per 100,000 (Supplementary Table S1). Rather than one cancer site, the advantage for Black married men was observed across multiple cancer sites. Among men, the IRR for never- versus ever-married status was 1.96 for Black men, 1.82 for Hispanic men, and 1.62 for both White and Asian/Pacific Islander men (Supplementary Table S3). Across racial and ethnic groups, marital status differences were largest among Black men and consistently large among women of all races, with IRRs clustering around 1.90 to 1.94, indicating greater population-level disadvantage associated with never-married status in these groups.

Among men, there was significant heterogeneity in the magnitude of marital status differences by race/ethnicity (*P* = 0.008 for interaction). Among women, the interaction between race and marital status was not statistically significant overall (*P* = 0.278), indicating that the effect of marital status on cancer incidence was relatively consistent across racial/ethnic groups.

Cancer incidence differences by marital status also varied significantly by cancer site (Supplementary Table S2). The strongest associations were observed for anal (IRR 5 in men; 2.5 in women), esophageal (2.4; 2.7), cervical (2.6), ovarian (2.4), uterine (2.4), HCC (2.3; 2.3), bladder (2.3 females only), lung (2.1; 2.1), and colorectal (2.1 females only) cancers. In contrast, relatively modest differences by marital status were seen for thyroid (IRRs 1.2; 1.6), skin melanoma (1.4; 1.6), prostate (1.5), testis (1.6), kidney (1.6 in males only), and brain (1.6 in males only) cancers. The female breast cancer IRR was 1.7 with no difference between the two most common types: triple-negative breast cancers and hormone-positive HER2-negative cancers. Prostate cancer diagnosed at lower PSA levels (PSA1), which likely reflects screening intensity rather than increased risk, showed a comparatively modest association with marital status (IRR = 1.36; 95% CI, 1.24–1.49), compared with PSA2 (IRR = 1.90; 95% CI, 1.85–1.96) and PSA3 (IRR = 2.62; 95% CI, 2.43–2.83). Similarly, thyroid cancer, another malignancy strongly influenced by detection and screening practices, showed one of the smallest differences between ever- and never-married adults (IRR = 1.23 in men; 1.60 in women). Within cancer sites, further racial and ethnic variation in marital status differences was also observed (Table 2).

**Table 2.** IRRs of never-married vs. ever-married adults ≥30 years for six screenable cancer sites by race/ethnicity and sex, SEER 12 states combined, 2015–2022.

Cancer site	Race/ethnicity	Males	Females
		IRR (95% CI)	IRR (95% CI)
Breast	White	—	1.67 (1.52–1.84)
​	Black	—	1.71 (1.58–1.86)
​	Hispanic	—	1.71 (1.56–1.87)
​	Asian/Pacific Islander	—	1.79 (1.64–1.97)
Cervix	White	—	2.90 (2.60–3.23)
​	Black	—	2.64 (2.46–2.83)
​	Hispanic	—	3.01 (2.72–3.33)
​	Asian/Pacific Islander	—	2.21 (2.03–2.41)
Colorectal	White	1.87 (1.69–2.06)	2.04 (1.85–2.24)
​	Black	2.16 (1.98–2.35)	2.25 (2.18–2.33)
​	Hispanic	1.92 (1.69–2.17)	2.11 (1.92–2.32)
​	Asian/Pacific Islander	1.90 (1.72–2.11)	2.26 (2.08–2.45)
HCC	White	2.43 (2.20–2.69)	2.59 (2.27–2.94)
​	Black	3.20 (3.04–3.35)	2.69 (2.27–3.18)
​	Hispanic	2.72 (2.61–2.83)	2.59 (2.43–2.76)
​	Asian/Pacific Islander	1.97 (1.84–2.11)	1.87 (1.66–2.10)
Lung	White	2.02 (1.84–2.21)	2.15 (2.01–2.29)
​	Black	2.78 (2.69–2.88)	2.60 (2.51–2.69)
​	Hispanic	2.38 (2.12–2.67)	2.24 (2.12–2.37)
​	Asian/Pacific Islander	1.92 (1.86–2.01)	1.89 (1.66–2.15)
Prostate	White	1.36 (1.26–1.48)	—
​	Black	1.67 (1.48–1.88)	—
​	Hispanic	1.66 (1.51–1.82)	—
​	Asian/Pacific Islander	1.53 (1.42–1.65)	—

All negative binomial models demonstrated good fit based on deviance and information criteria. Sensitivity analyses restricted to complete case data yielded IRRs nearly identical to those from imputed datasets, confirming that imputation did not materially affect study findings.

## Discussion

Marital status may be a powerful and underrecognized social determinant of cancer risk. To our knowledge, this is the largest comprehensive, population-based study of cancer incidence by marital status in the United States. We found that never-married adults, both women and men, experienced substantially higher cancer incidence across nearly all major cancer sites, racial/ethnic groups, and age groups compared with ever-married individuals. Most site-specific IRRs exceeded 1.5, and several approached or surpassed 2. These findings provide a contemporary update to a historically understudied topic and underscore the significance of legal marital status *per se* as a structural determinant of cancer risk.

Never-married men had 68% higher cancer incidence, and never-married women had 83% higher incidence compared with their ever-married counterparts. Age-stratified analyses using broader age groupings (30–54 vs. ≥55) revealed that associations were strongest among adults ages ≥55 years, suggesting that differences associated with marital status may accumulate over the life course. In contrast, smaller IRRs among adults ages 30 to 54 years likely reflect selection processes, whereby individuals with more favorable baseline health, behaviors, or resources are more likely to marry.

Marital status is often treated as a background demographic variable. Yet, our findings suggest that it may function as a social exposure that captures dimensions of cancer risk not fully explained by race, age, or socioeconomic status (SES). It may serve both as a marker of cumulative social advantage and as a multifactorial exposure encompassing behavioral, psychosocial, and healthcare-related factors, such as sexual behavior, parity, tobacco and alcohol use, diet, and engagement with preventive care (26–34). The IRRs associated with never-married status were frequently larger than those observed for race/ethnicity or SES, highlighting its epidemiologic relevance. Given that approximately 20% of adults ages ≥30 years are never-married, the population-level impact of this disparity is substantial.

Site-specific patterns offer further insight into potential mechanisms linking marital status and cancer. Never-married individuals had the highest excess risks for HPV-related cancers, with IRRs exceeding five for anal cancer in men and approaching three for cervical cancer in women, with findings consistent with differences in sexual behavior, HPV exposure, and screening uptake (14, 44–46). Specifically for anal cancer in men, these patterns may reflect the clustering of unmeasured risk factors such as male–male sexual contact and HIV infection, which are more prevalent among never-married men (47).

Tobacco-related cancers, including lung and esophageal cancers, were also more common among the never-married, reflecting known associations with smoking and alcohol use (26–28, 34). Elevated rates of stomach, colorectal, and HCC cancers further implicate behavioral and environmental risk factors. Among women, the higher incidence of endometrial and ovarian cancers among the never-married supports reproductive mechanisms, including nulliparity (48–51).

Differences across racial and ethnic groups further illustrate how both biological and social pathways shape risk. For example, the difference in HCC incidence between ever- and never-married individuals was smaller among Asian/Pacific Islander adults, likely due to the predominance of perinatally acquired hepatitis B virus, a less modifiable risk factor. In contrast, HCC in non-Asian populations is more often linked to hepatitis C and alcohol use (52), factors more susceptible to behavioral and social influences.

For behaviorally influenced cancers with known direct causes, such as liver and lung cancers, disparities between never- and ever-married individuals were particularly pronounced among Black and Hispanic adults. These elevated IRRs likely reflect a combination of mechanisms: stronger selection into marriage in groups with lower marriage prevalence (35–37) and the acquisition of healthier behaviors, increased healthcare engagement, and social support associated with marital status over the life course (26–31, 34). The same pattern is evident in overall cancer incidence: never-married Black men had the highest rates of all groups, yet married Black men had lower rates than married White men. In populations facing structural barriers to marriage, including systemic racism, economic exclusion, and disproportionate incarceration (22, 53), those who marry may be a more selected group in terms of health and stability, which could strengthen the observed protective effects of marriage.

Among women, IRRs clustered around 1.9 across all racial and ethnic groups, indicating consistently elevated cancer incidence among never-married women. This pattern contrasts with the greater heterogeneity observed among men and suggests that marital status differences in cancer incidence are more uniform across racial groups in women. Notably, these patterns do not support the long-standing assumption that the health benefits of marriage are greater for men than women (7, 8, 12). Instead, they suggest that marital status is at least as salient a social stratifier of cancer risk among women as among men, highlighting the need to reconsider sex-specific pathways linking marital status to cancer incidence, particularly for cancers shaped by cumulative social and behavioral exposures.

From an epidemiology perspective, the magnitude of marital status disparities offers insight into how modifiable a cancer’s etiology may be, indirectly revealing which cancers are more responsive to social and behavioral influences and potentially more targetable by public health. This was most evident for HPV-related, tobacco-related, and reproductive cancers, which showed the largest differences in cancer risk. In contrast, cancers such as breast, prostate, and thyroid cancers exhibited weaker associations, suggesting less modifiable etiologies, potentially due to biological factors, early-life, or unidentified exposures.

This study has limitations. Legal marital status is a heterogeneous administrative classification that does not directly measure social support, partnership quality, cohabitation, or relational stability (54). Individuals in long-term cohabiting relationships may experience levels of emotional and instrumental support similar to married individuals yet are classified as never-married in registry and census data. Conversely, individuals in strained or abusive marriages may not experience protective social benefits despite being categorized as married (55). Therefore, legal marriage should not be interpreted as a direct proxy for social support but rather as a structural and institutional marker that may correlate with broader social and behavioral patterns influencing cancer risk.

We acknowledge that the “ever-married” category aggregates heterogeneous marital histories (currently married, divorced, and widowed). Parsing these groups would require time-varying marital trajectories and careful attention to selection and competing risks. For instance, widowed individuals may seem to have lower cancer incidence because they represent survivors of long unions and face competing mortality risks, making that contrast etiologically distinct from the never- versus ever-married comparison addressed here. Our prespecified focus therefore remained on the never- versus ever-married contrast to provide a clear, comparable benchmark for surveillance and policy. Detailed analyses by marital subcategories constitute a separate research question that warrants dedicated study. SEER and the ACS do not capture same-sex partnerships or sexual orientation; therefore, we were unable to examine cancer incidence differences by sexual minority status or disentangle its potential effects from legal marital status. Marital status was assessed only at diagnosis, precluding analysis of temporal changes. We also lacked individual-level data on income, education, parity, and health behaviors, which may lie on the causal pathway between marital status and cancer risk (56). These variables are plausibly both antecedents and consequences of marriage, but in their downstream role, are better conceptualized as mediators rather than confounders. Adjusting for them would likely result in overadjustment and obscure the total effect of marital status on cancer incidence. Finally, although many studies have examined cancer mortality by marital status (57), such comparisons are limited by misclassification on death certificates and changes in marital status between diagnosis and death. In contrast, incidence-based analyses provide a more stable and interpretable measure of the relationship between marital status and cancer risk.

Taken together, these findings highlight marital status as a prominent and consistent social stratifier of cancer incidence in the contemporary United States. Despite the limitations, this study offers a timely and robust contribution to understanding cancer disparities. In the context of declining marriage rates, delayed or forgone childbearing, and shifting social norms (22–25), never-married adults may represent a vulnerable group for cancer prevention and early detection. Supporting individuals who wish to marry or have children, by addressing barriers such as economic insecurity, housing instability, and structural racism, may have downstream implications for cancer risk. Public health efforts that acknowledge these social determinants may help reduce disparities in cancer incidence and promote more equitable outcomes. Integrating marital status into cancer surveillance and risk stratification frameworks may enhance identification of at-risk populations and inform more targeted prevention strategies.

## Supplementary Material

Supplementary Table S1 Age-adjusted incidence rates with 95% confidence intervals of all cancers combined among adults ≥30 years, by race/ethnicity, sex, and marital status, SEER 12 states combined, 2015-2022.

Supplementary Table S2 Cancer site-specific incidence rate ratios (IRRs) and 95% confidence intervals among never-married vs. ever-married adults ≥ 30 years and corresponding age- adjusted incidence rates stratified by sex, SEER 12 states combined, 2015-2022.

Supplementary Table S3 Incidence rate ratios of never-married vs. ever-married adults ≥30 years by race/ethnicity and sex, SEER 12 states combined, 2015-2022.

Supplementary Table S4 Age-specific incidence rate ratios of never-married vs ever-married adults ≥ 30 years by sex, SEER 12 states combined, 2015-2022.

Supplementary Table S5 Incidence rate ratios of never-married vs ever-married adults ≥ 30 years for six screenable cancer sites by stage at diagnosis and sex, SEER 12 states combined, 2015-2022.

Supplementary Figure S1 Incidence rate ratio of never-married vs. ever-married for six screenable cancers by stage and sex, SEER 12 states combined, 2015-2022.

## Data Availability

The cancer incidence data underlying this article are available from the SEER website at https://seer.cancer.gov/. The population denominators underlying this article are available from the US Census Bureau’s ACS at https://doi.org/10.18128/D010.V16.0. All other data are available in the main article, supplemental files, or upon request from the corresponding author.
