# Supplementary material for: Marriage and Cancer Risk: A Contemporary Population-Based Study Across Demographic Groups and Cancer Types
Source: Cancer Res Commun. 2026 Apr 8;6(4):783–91. doi: 10.1158/2767-9764.CRC-25-0814 (PMC13058905; doi:10.1158/2767-9764.CRC-25-0814)
Supplement: Supplementary Table S1 — Age-adjusted incidence rates with 95% confidence intervals of all cancers combined among adults ≥30 years, by race/ethnicity, sex, and marital status, SEER 12 states combined, 2015-2022. [file crc-25-0814_supplementary_table_s1_suppst1.docx]

**Supplementary Table S1.** Age-adjusted incidence rates with 95% confidence intervals of all cancers combined among adults ≥30 years, by race/ethnicity, sex, and marital status, SEER 12 states combined, 2015-2022.

| Incidence Rate (95% CI)^a,b^ | Males | | Females | |  |  |
| --- | --- | --- | --- | --- | --- | --- |
| Race/ Ethnicity | Ever-married | Never-married | Ever-married | Never-married | |  |
| ALL COMBINED^c^ | 756.0 (754.9 to 757.2) | 1397.8 (1392.4 to 1403.1) | 639.1 (638.1 to 640.1) | 1267.8 (1263.5 to 1272.0) | |  |
| White | 836.2 (834.7 to 837.7) | 1454.3 (1447.4 to 1461.3) | 712.0 (710.6 to 713.4) | 1467.6 (1461.0 to 1474.3) | |  |
| Black | 752.6 (748.8 to 756.5) | 1600.6 (1585.8 to 1615.4) | 545.5 (542.6 to 548.5) | 1137.9 (1129.2 to 1146.6) | |  |
| Hispanic | 551.2 (548.6 to 553.9) | 1125.7 (1113.3 to 1138.2) | 508.0 (505.7 to 510.2) | 1057.2 (1048.4 to 1066.0) | |  |
| Asian/Pacific Islander | 476.7 (473.9 to 479.5) | 884.2 (868.0 to 900.7) | 477.2 (474.7 to 479.7) | 961.6 (948.9 to 974.4) | |  |
| ^a^ CI = confidence interval  ^b^ Incidence rates per 100,000 population, age-adjusted to the 2000 U.S. standard population  ^c^ All combined race category includes individuals of other or not specified racial/ethnic groups | | | | | | |
