# Supplementary material for: Marriage and Cancer Risk: A Contemporary Population-Based Study Across Demographic Groups and Cancer Types
Source: Cancer Res Commun. 2026 Apr 8;6(4):783–91. doi: 10.1158/2767-9764.CRC-25-0814 (PMC13058905; doi:10.1158/2767-9764.CRC-25-0814)
Supplement: Supplementary Table S2 — Cancer site-specific incidence rate ratios (IRRs) and 95% confidence intervals among never-married vs. ever-married adults ≥ 30 years and corresponding age- adjusted incidence rates stratified by sex, SEER 12 states combined, 2015-2022. [file crc-25-0814_supplementary_table_s2_suppst2.docx]

**Supplementary Table S2.** Cancer site-specific incidence rate ratios (IRRs) and 95% confidence intervals among never-married vs. ever-married adults ≥ 30 years and corresponding age- adjusted incidence rates stratified by sex, SEER 12 states combined, 2015-2022.

|  | Male | | | | | Female | | | | | | |  |
| --- | --- | --- | --- | --- | --- | --- | --- | --- | --- | --- | --- | --- | --- |
| Cancer Site | | IRR (95% CI)^a^ | | Incidence (Ever- married)^b^ | | | IRR (95% CI)^a^ | | Incidence  (Ever- married)^b^ | |  |  |  |
| ALL COMBINED | | 1.68 (1.53 to 1.84) | | - | | | 1.85 (1.68 to 2.03) | | - | |  |  |  |
| Anus | | | 5.04 (4.39 to 5.78) | | 1.8 | | | 2.50 (2.30 to 2.72) | | 3.5 | | | |
| Breast | | | - | | - | | | 1.69 (1.59 to 1.78) | | 205.4 | | | |
| HR+/HER2-^c^ | | | - | | - | | | 1.68 (1.59 to 1.78) | | 143.3 | | | |
| TNBC^c^ | | | - | | - | | | 1.67 (1.58 to 1.77) | | 20.5 | | | |
| Bladder | | | 1.80 (1.72 to 1.89) | | 52.4 | | | 2.32 (2.21 to 2.45) | | 12.4 | | | |
| Brain | | | 1.56 (1.47 to 1.65) | | 9.8 | | | 1.84 (1.73 to 1.95) | | 6.6 | | | |
| Cervix | | | - | | - | | | 2.64 (2.42 to 2.87) | | 9.6 | | | |
| Colorectal | | | 1.89 (1.78 to 2.01) | | 64.2 | | | 2.10 (2.00 to 2.20) | | 49.2 | | | |
| Esophagus | | | 2.39 (2.27 to 2.53) | | 10.6 | | | 2.74 (2.54 to 2.96) | | 2.5 | | | |
| HCC^c^ | | | 2.31 (2.08 to 2.56) | | 16.8 | | | 2.32 (2.08 to 2.58) | | 5.2 | | | |
| Hodgkin Lymphoma | | | 1.69 (1.53 to 1.88) | | 3.0 | | | 1.83 (1.64 to 2.04) | | 2.1 | | | |
| Kidney | | | 1.59 (1.52 to 1.66) | | 36.1 | | | 1.84 (1.75 to 1.93) | | 17.1 | | | |
| Leukemia | | | 1.66 (1.56 to 1.78) | | 26.1 | | | 1.88 (1.78 to 1.99) | | 15.2 | | | |
| Lung | | | 2.13 (1.99 to 2.28) | | 85.2 | | | 2.08 (1.94 to 2.23) | | 69.3 | | | |
| Multiple Myeloma | | | 1.69 (1.62 to 1.76) | | 14.1 | | | 2.00 (1.92 to 2.08) | | 9.0 | | | |
| Non-Hodgkin Lymphoma | | | 1.69 (1.61 to 1.78) | | 35.3 | | | 1.98 (1.93 to 2.03) | | 24.2 | | | |
| Oral | | | 1.94 (1.77 to 2.12) | | 26.1 | | | 1.96 (1.83 to 2.10) | | 9.7 | | | |
| Ovary | | | - | | - | | | 2.37 (2.23 to 2.51) | | 14.9 | | | |
| Pancreas | | | 1.87 (1.79 to 1.95) | | 24.1 | | | 2.04 (1.97 to 2.12) | | 18.6 | | | |
| Prostate | | | 1.48 (1.34 to 1.61) | | 193.3 | | | - | | - | | | |
| PSA1^c^ | | | 1.36 (1.24 to 1.49) | | 132.2 | | | - | | - | | | |
| PSA2^c^ | | | 1.90 (1.85 to 1.96) | | 11.0 | | | - | | - | | | |
| PSA3^c^ | | | 2.62 (2.43 to 2.83) | | 13.0 | | | - | | - | | | |
| Skin Myeloma | | | 1.41 (1.33 to 1.50) | | 48.2 | | | 1.55 (1.44 to 1.68) | | 28.6 | | | |
| Stomach | | | 1.85 (1.77 to 1.95) | | 14.9 | | | 1.98 (1.87 to 2.09) | | 8.5 | | | |
| Testicle | | | 1.60 (1.46 to 1.75) | | 6.3 | | | - | | - | | | |
| Thyroid | | | 1.23 (1.16 to 1.31) | | 12.2 | | | 1.60 (1.50 to 1.71) | | 30.7 | | | |
| Uterine | | | - | | - | | | 2.35 (2.16 to 2.56) | | 41.3 | | |  |
| ^a^ CI = confidence interval; IRR = incidence rate ratio  ^b^ Incidence rates per 100,000 population, age-adjusted to the 2000 U.S. standard population  ^c^ HCC = hepatocellular carcinoma; TNBC = triple negative breast cancer; HR+/HER2- = hormone receptor positive/ human epidermal growth factor receptor 2 negative; PSA 1-3 = prostate specific antigen 1-3 | | | | | | | | | | | |  |  |
