# Supplementary material for: Marriage and Cancer Risk: A Contemporary Population-Based Study Across Demographic Groups and Cancer Types
Source: Cancer Res Commun. 2026 Apr 8;6(4):783–91. doi: 10.1158/2767-9764.CRC-25-0814 (PMC13058905; doi:10.1158/2767-9764.CRC-25-0814)
Supplement: Supplementary Table S3 — Incidence rate ratios of never-married vs. ever-married adults ≥30 years by race/ethnicity and sex, SEER 12 states combined, 2015-2022. [file crc-25-0814_supplementary_table_s3_suppst3.docx]

**Supplementary Table S3.** Incidence rate ratios of never-married vs. ever-married adults ≥30 years by race/ethnicity and sex, SEER 12 states combined, 2015-2022.

|  | Males | Females |
| --- | --- | --- |
| Race/ Ethnicity | IRR (95% CI)^a^ | IRR (95% CI)^a^ |
| ALL COMBINED^b^ | 1.68 (1.53 to 1.84) | 1.85 (1.68 to 2.03) |
| White | 1.62 (1.33 to 1.96) | 1.90 (1.55 to 2.32) |
| Black | 1.96 (1.58 to 2.44) | 1.92 (1.56 to 2.37) |
| Hispanic | 1.82 (1.50 to 2.22) | 1.94 (1.60 to 2.35) |
| Asian/Pacific Islander | 1.62 (1.32 to 2.00) | 1.90 (1.52 to 2.34) |
| ^a^ CI = confidence interval; IRR = incidence rate ratio  ^b^ All combined race category includes individuals of other or not specified racial/ethnic groups | | |
