# Supplementary material for: Marriage and Cancer Risk: A Contemporary Population-Based Study Across Demographic Groups and Cancer Types
Source: Cancer Res Commun. 2026 Apr 8;6(4):783–91. doi: 10.1158/2767-9764.CRC-25-0814 (PMC13058905; doi:10.1158/2767-9764.CRC-25-0814)
Supplement: Supplementary Table S4 — Age-specific incidence rate ratios of never-married vs ever-married adults ≥ 30 years by sex, SEER 12 states combined, 2015-2022. [file crc-25-0814_supplementary_table_s4_suppst4.docx]

**Supplementary Table S4.** Age-specific incidence rate ratios of never-married vs ever-married adults ≥ 30 years by sex, SEER 12 states combined, 2015-2022.

|  | Males | Females |
| --- | --- | --- |
| Age group (years) | IRR (95% CI)^a^ | IRR (95% CI)^a^ |
| 30-34 | 1.11 (1.08 to 1.14) | 1.11 (1.09 to 1.14) |
| 35-39 | 1.29 (1.26 to 1.32) | 1.38 (1.36 to 1.41) |
| 40-44 | 1.43 (1.40 to 1.46) | 1.52 (1.49 to 1.54) |
| 45-49 | 1.67 (1.65 to 1.70) | 1.72 (1.70 to 1.74) |
| 50-54 | 1.77 (1.75 to 1.80) | 1.90 (1.88 to 1.92) |
| 55-59 | 1.89 (1.87 to 1.90) | 2.16 (2.14 to 2.18) |
| 60-64 | 1.91 (1.89 to 1.92) | 2.20 (2.18 to 2.22) |
| 65-69 | 1.96 (1.94 to 1.98) | 2.23 (2.21 to 2.25) |
| 70-74 | 1.99 (1.97 to 2.01) | 2.23 (2.21 to 2.25) |
| 75-79 | 1.96 (1.94 to 1.98) | 2.18 (2.16 to 2.21) |
| 80-84 | 1.86 (1.83 to 1.89) | 2.01 (1.98 to 2.04) |
| 85+ | 1.60 (1.57 to 1.63) | 1.73 (1.71 to 1.76) |
| ^a^ CI = confidence interval; IRR = incidence rate ratio | | |
