# Supplementary material for: Marriage and Cancer Risk: A Contemporary Population-Based Study Across Demographic Groups and Cancer Types
Source: Cancer Res Commun. 2026 Apr 8;6(4):783–91. doi: 10.1158/2767-9764.CRC-25-0814 (PMC13058905; doi:10.1158/2767-9764.CRC-25-0814)
Supplement: Supplementary Table S5 — Incidence rate ratios of never-married vs ever-married adults ≥ 30 years for six screenable cancer sites by stage at diagnosis and sex, SEER 12 states combined, 2015-2022. [file crc-25-0814_supplementary_table_s5_suppst5.docx]

**Supplementary Table S5.** Incidence rate ratios of never-married vs ever-married adults ≥ 30 years for six screenable cancer sites by stage at diagnosis and sex, SEER 12 states combined, 2015-2022.

|  |  | Males | Females |
| --- | --- | --- | --- |
| Cancer Site | Stage at Diagnosis | IRR (95% CI)^a^ | IRR (95% CI)^a^ |
| Breast | Localized | - | 1.54 (1.43 to 1.67) |
|  | Regional | - | 1.68 (1.52 to 1.86) |
|  | Distant | - | 2.43 (2.18 to 2.71) |
| Cervix | Localized | - | 2.49 (2.22 to 2.79) |
|  | Regional | - | 3.14 (3.01 to 3.28) |
|  | Distant | - | 3.68 (3.46 to 3.93) |
| Colorectal | Localized | 1.69 (1.52 to 1.86) | 1.92 (1.75 to 2.10) |
|  | Regional | 1.82 (1.63 to 2.03) | 2.04 (1.87 to 2.22) |
|  | Distant | 2.24 (1.98 to 2.54) | 2.28 (2.07 to 2.53) |
| HCC^b^ | Localized | 2.30 (2.11 to 2.51) | 2.74 (2.58 to 2.90) |
|  | Regional | 2.55 (2.29 to 2.84) | 2.77 (2.53 to 3.04) |
|  | Distant | 3.23 (3.07 to 3.39) | 3.07 (2.80 to 3.38) |
| Lung | Localized | 1.84 (1.65 to 2.04) | 2.00 (1.94 to 2.05) |
|  | Regional | 2.01 (1.86 to 2.18) | 2.09 (2.03 to 2.16) |
|  | Distant | 2.24 (2.07 to 2.42) | 2.12 (1.98 to 2.26) |
| Prostate | Localized | 1.50 (1.42 to 1.56) | - |
|  | Regional | 1.39 (1.32 to 1.46) | - |
|  | Distant | 2.58 (2.39 to 2.79) | - |
| ^a^ CI = confidence interval; IRR = incidence rate ratio  ^b^ HCC = hepatocellular carcinoma | | | |
