# Supplementary figures and images for: Marriage and Cancer Risk: A Contemporary Population-Based Study Across Demographic Groups and Cancer Types
Source: Cancer Res Commun. 2026 Apr 8;6(4):783–91. doi: 10.1158/2767-9764.CRC-25-0814 (PMC13058905; doi:10.1158/2767-9764.CRC-25-0814)

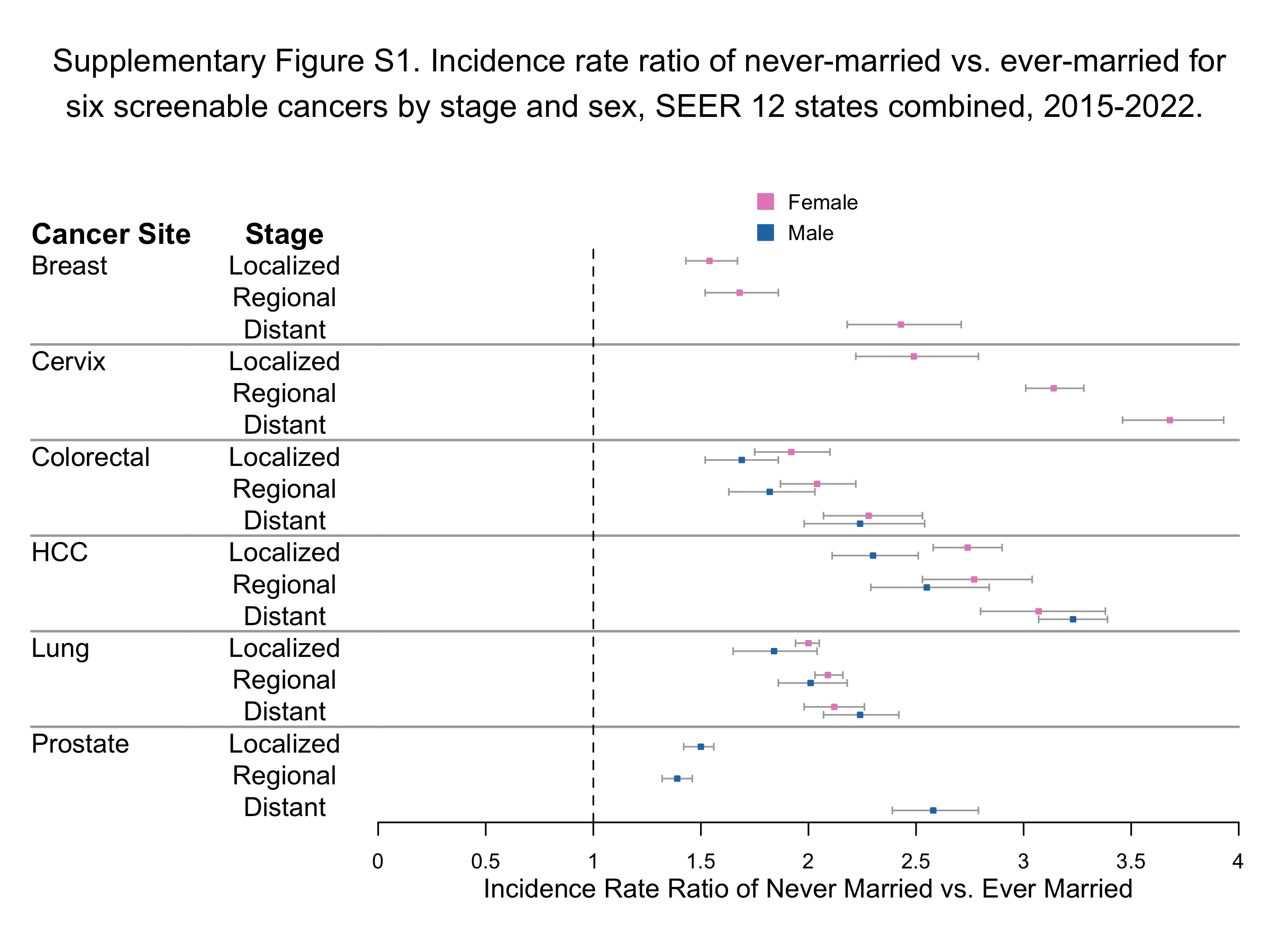

Supplement: Supplementary Figure S1 — Incidence rate ratio of never-married vs. ever-married for six screenable cancers by stage and sex, SEER 12 states combined, 2015-2022. [file crc-25-0814_supplementary_figure_s1_suppsf1.png]
